# Managing obstructive sleep apnoea in children: the role of craniofacial morphology

**DOI:** 10.6061/clinics/2016(11)08

**Published:** 2016-11

**Authors:** Maria Fernanda Rabelo Bozzini, Renata Cantisani Di Francesco

**Affiliations:** I Faculdade de Medicina da Universidade de São Paulo, São Paulo/SP, Brazil; II Faculdade de Medicina da Universidade de São Paulo, Departamento de Otorrinolaringologia, São Paulo/SP, Brazil

**Keywords:** Craniofacial Morphology, Sleep-disordered Breathing, Obstructive Sleep Apnoea Syndrome, Polysomnography, Children

## Abstract

Obstructive sleep apnoea syndrome is a type of sleep-disordered breathing that affects 1 to 5% of all children. Pharyngeal and palatine tonsil hypertrophy is the main predisposing factor. Various abnormalities are predisposing factors for obstructive sleep apnoea, such as decreased mandibular and maxillary lengths, skeletal retrusion, increased lower facial height and, consequently, increased total anterior facial height, a larger cranio-cervical angle, small posterior airway space and an inferiorly positioned hyoid bone. The diagnosis is based on the clinical history, a physical examination and tests confirming the presence and severity of upper airway obstruction. The gold standard test for diagnosis is overnight polysomnography. Attention must be paid to identify the craniofacial characteristics. When necessary, children should be referred to orthodontists and/or sleep medicine specialists for adequate treatment in addition to undergoing an adenotonsillectomy.

## INTRODUCTION

Obstructive sleep apnoea syndrome (OSAS) is defined as a type of sleep-disordered breathing (SDB) characterized by partial and/or complete upper airway obstruction that affects normal ventilation 1. Decreased quality of life and behavioural, psychiatric, neurocognitive, cardiovascular, metabolic, endocrine, and growth abnormalities are several of the complications associated with this syndrome 2.

Obstructive sleep apnoea (OSA) is estimated to affect 1 to 5% of all children 3, and its incidence peaks are between 3 and 8 years of age 4. Multiple components are involved in SDB in children. Anatomical, craniofacial and neuromuscular factors, excess lymphoid tissues and airway inflammation are cited as the most critical components 2.

Adenoid and tonsil hypertrophy are more prevalent between 3 and 6 years of age 5, and these enlargements are strongly related to OSAS in children 1. Snoring is one of the most often reported symptoms of SDB in paediatric populations, and its prevalence ranges from 1.5 to 27.6% for different studies and populations 2.

### SDB and Craniofacial Morphology

At birth, the face is approximately 40% of the adult size, and it increases to 65% by 3 years of age. Facial growth is only completed after puberty. Facial growth is influenced mainly by genetic factors, but environmental aspects such as the breathing pattern may also contribute to growth 6.

Nasal breathing in children may induce a correction of craniofacial growth and the adequate development of other functions, such as chewing and swallowing 7. To promote breathing in the presence of nasal obstructions, the head and mandible position and the tonicity of the tongue and orofacial muscles are altered. When they persist, these changes modify the equilibrium of muscle pressure on the facial bones and teeth and induce morphological dentoskeletal modifications 8.

Various craniofacial deformities are predisposing factors, including decreased mandibular and maxillary lengths, skeletal retrusion, increased lower facial height and, consequently, increased total anterior facial height, a larger cranio-cervical angle, decreased posterior airway space and an inferiorly positioned hyoid bone 1,5,.

A positive correlation exists between the severity of the breathing pattern as measured by the apnoea and hypo-apnoea index and changes in dentofacial development. Dental arch abnormalities can be explained by long-term changes in the head, mandible and tongue positions 10. The most common dental and skeletal alterations are transverse maxillary deficiency, open bite, large overjet, retro-inclined lower incisors and protruding upper incisors 9. The prevalence of posterior crossbite is significantly more frequent in mouth breathers (49%) than in nose breathers (26%) 14, and children with OSA commonly have shorter inter-molar and inter-canine distances 15.

In a recent meta-analysis, Katyal 16 stated that “there is no evidence for a direct causal relationship between craniofacial structure and paediatric SDB and there is lack of intensive evidence on the critical degree of obstruction, or how long it may occur to affect growth”.

### Diagnosis

Children with OSAS often have cognitive problems, disturbed sleep, delayed learning, social performance disorders, bruxism and enuresis. The diagnosis is based on clinical history, a physical examination and tests confirming the presence and severity of the obstruction 9.

The gold standard for the diagnosis is overnight polysomnography, which is currently the only reliable diagnostic modality that can discern SDB from primary snoring 13. Craniofacial anatomy plays a role in OSAS, and screening for anatomical abnormalities is an important tool in its diagnosis.

Advanced technologies, such as computed tomography and magnetic resonance imaging, are currently used to evaluate the anatomical characteristics of the upper airway and craniofacial structures. However, the traditional cephalometric method is the most practical technique and is frequently used in clinical practice due to its easy access, low cost and low radiation exposure 11.

One misleading problem in assessing craniofacial morphology using a lateral cephalogram is that patients are conscious, in an upright position and have occluded teeth. Paediatric SDB typically worsens in the supine position when muscle hypotony occurs while sleeping. Further investigations are required to address the relationship between craniofacial morphology and paediatric SDB in all three dimensions 16.

### Treatment

The treatment is based upon the severity of obstructive apnoea. The practice guidelines established by the American Academy of Pediatricians proposes an adenotonsillectomy as the first line of therapy for childhood OSAS 4,17,18. Several meta-analyses have reported that an adenotonsillectomy leads to significant improvements in most cases of paediatric OSAS 4,19; however, the success rates are highly variable and range from 24 to 100% 20-22.

Changing the breathing pattern from oral to nasal early in adolescence may benefit the craniofacial dimensions during growth 23. Previous studies have provided evidence for quality of life gains promptly after surgery, mainly for patients with more severe obstructive sleep disorders, and this effect is not significantly affected by gender, age or adiposity 24. An adenotonsillectomy can also efficiently improve several dental malocclusions, which benefits patients during their growth phase. In addition, this technique is mandatory for proper occlusal development 25.

However, the structural problems in many children with SDB are not fully solved after an adenotonsillectomy, and a risk exists for a slow but progressive reappearance of OSA symptoms 21. An adenotonsillectomy is associated with significant improvements in most children, but approximately 20 to 30% may exhibit residual symptoms; this percentage can increase to 70% in patients with a high apnoea-hypopnoea index on preoperative polysomnography 4,17.

The recurrence is typically not immediate and may occur during the pubertal period and after the mean age at which dentoskeletal development reaches approximately 90% of its adult final growth 26. Mouth-breathing children may exhibit a dolichofacial pattern due to the mandibular plane inclination, the gonial angle and the increased posterior facial height 27. The presence of residual OSA in a large number of patients following an adenotonsillectomy is a significant public health concern and calls for further studies focusing on individual patient factors that may predict the success rates and additional therapies that may improve surgery results in these patients 19.

These findings raise important issues regarding the efficacy of an adenotonsillectomy and further suggest that overnight polysomnography should be routinely implemented 19-20. The primary justification for surgery must be to open the airways such that physiological nose breathing is possible. If the clinical follow-up after surgery shows that the child maintains mouth breathing, it is important to examine the child for nasal congestion, including septal deviations or allergic rhinitis. Adjuvant therapy, such as orthodontic maxillary expansion and/or functional training, must also be considered 28. OSA appears to be more severe in boys than in girls due to the craniofacial morphology, which may be relevant for preventing sleep apnoea in adult males. The long-term follow-up of OSA in boys is mandatory for preventing the disease in adulthood 29.

Given the impact on a child’s health, determination of these predictor factors is important to prevent and manage SDB. If a health professional notices signs and symptoms SDB, the young patient should be referred to a sleep medicine specialist and to an orthodontist if dentoskeletal abnormalities are present 30.

In adults, continuous positive airway pressure therapy is the first line of treatment for patients with OSAS. This procedure prevents upper airway collapse and relieves symptoms but has poor compliance. An alternative is maxillomandibular advancement, which enlarges the pharyngeal space and is often performed with genioglossus advancement to decrease the amount of tongue blockage during sleep 31. The success rates and long-term stability of outcomes confirm the efficacy of maxillomandibular advancement; however, continuous follow-up is necessary to control patient lifestyle and detect possible relapses 32. The choice of this surgery should follow the failure of other treatment procedures because a longer recovery period is necessary than for other options.

Further standardization of research methods is recommended, including the establishment and acceptance of valid definitions for normal breathing, SDB and OSA 16. Paediatricians and dentists should always ask parents about their young child’s nightly breathing/snoring and advise them to seek appropriate help for all children who snore and/or breathe orally.

Craniofacial morphology plays an important role in SDB in children. It is also associated with the recurrence of snoring and OSA after an adenotonsillectomy at later ages, including in teenagers and adults. Attention must be paid to identify these characteristics. When necessary, children should be referred to orthodontists and/or sleep medicine specialists for adequate treatment in addition to undergoing an adenotonsillectomy.

## AUTHOR CONTRIBUTIONS

Authors make substantial contributions to conception and design of this manuscript. Bozzini MF participated in drafting and writing this review article and together with Di Francesco RC revised it critically for important intellectual content. Both authors approved the final version of the manuscript to be submitted.
